# Plantar and dorsal approaches for excision of morton’s neuroma: a comparison study

**DOI:** 10.1186/s12891-022-05858-w

**Published:** 2022-10-06

**Authors:** Wenpeng Xu, Ning Zhang, Zhengxun Li, Yifan Wang, Xiucun Li, Yang Wang, Haipeng Si, Yong Hu

**Affiliations:** 1 Department of Orthopedics, Qilu Hospital of Shandong University, 250012 Jinan, China; 2 Department of Foot and Ankle Surgery, The second hospital of Shandong University, 250033 Jinan, China

**Keywords:** Level III, retrospective comparative case series, Dorsal approach, Morton’s neuroma, Neurectomy, Plantar approach

## Abstract

**Background:**

Morton’s neuroma is a painful enlargement of the plantar digital nerve between the metatarsal heads that causes pain of the forefoot. Several approaches have been used to treat Morton’s neuroma, each of them having distinct advantages and disadvantages.

**Objectives:**

The purpose of this study was to investigate and compare the clinical outcomes of neurectomy in the treatment of Morton’s neuroma through plantar and dorsal approaches.

**Materials and methods:**

A total of 20 patients with a mean age of 48.5 ± 13.0 years (range: 19–66 years) who underwent excision of a Morton’s neuroma that did not respond to conservative treatment were retrospectively analysed from June 2014 to June 2021. All the neurectomies were performed using a plantar or dorsal approach. Outcomes were evaluated using visual analogue scale (VAS) scores, American Orthopedic Foot and Ankle Society (AOFAS) scores, the Foot and Ankle Ability Measure (FAAM), and complications. The appearance index (AI) was also used to assess the influence of foot appearance on the quality of life after surgery.

**Results:**

Eight patients underwent neurectomy by the dorsal approach, and 12 patients underwent neurectomy by the plantar approach. The average follow-up time was 28.9 ± 12.9 months (range: 15–72 months). No statistically significant difference was found between the dorsal and plantar approach groups with respect to postoperative pain measured by the VAS score. The postoperative AOFAS scores and FAAM outcomes were not significantly different between the groups. The complications reported in the dorsal approach group were significantly less than those of the plantar group, mainly discomfort in wearing shoes. The AI of the plantar group and the dorsal group were significantly different.

**Conclusion:**

The excision of the Morton’s neuroma by both the dorsal and plantar approach resulted in satisfactory outcomes. However, the foot appearance after surgery by the plantar approach had less influence on the quality of life than that using the dorsal approach. Our recommendation is that surgeons should choose the approach they are most familiar with and with which they are most confident in performing. In addition, the plantar approach is recommended if the patient needs a better appearance.

## Introduction

Morton’s neuroma is a painful enlargement of the plantar digital nerve between the metatarsal heads, most frequently in the second and third intermetatarsal spaces, which cause pain of the forefoot [1]. Thomas Morton was the first describing the disease in 1876 and proposed that it was the result of digital nerve impingement between the metatarsal heads [2]. The aetiology and pathogenesis of Morton’s neuroma remain controversial [3]. One recent research on cadavers and living patients hypothesized that altered foot support and biomechanics may act on the interosseous muscles and increase the stiffness of the dorsal fascia, particularly at the point where these muscles are inserted. Chronic rigidity of this fascia causes increased stiffness of the inter-metatarsal space. Neuroma may be considered as the final manifestation [4]. The mean age at the time of symptoms ranges from forty-five to fifty years, and women are more frequently affected [5]. Axial compression may be accompanied by a demonstrable painful click known as Mulder’s sign. Ultrasound and magnetic resonance imaging (MRI) scans are used to diagnose Morton’s neuroma, but the clinical history and examination remain the most sensitive and specific methods to diagnose correctly a neuroma [6, 7].

Conservative management is recommended at first, including lifestyle modifications (avoidance of tight-fitting shoes, orthotics, and local infiltrations of corticosteroids), as well as mobilisation and manipulation techniques. Surgical intervention is recommended after the failure of conservative therapy [8]. Neurectomy is a standard operating procedure in the treatment of Morton’s neuroma, although many others were reported [9]. Indeed, several surgical approaches have been used to treat Morton’s neuroma. The plantar longitudinal approach and the dorsal approach are the most used, and they have distinct advantages and disadvantages [10–13].

To our knowledge, only few studies are available to compare the operative outcomes using the dorsal and plantar approach. The purpose of this retrospective study was to investigate and compare the clinical outcomes of neurectomy in the treatment of Morton’s neuroma by plantar and dorsal approaches.

## Materials and methods

Twenty patients in our institute who underwent excision of a Morton’s neuroma that was unresponsive to conservative treatment were retrospectively analysed from June 2014 to June 2021. All patients were diagnosed by one orthopaedic surgeon based on medical history, physical examination, MRI, and ultrasound. Eight patients received 9 neurectomies by the dorsal approach, and 12 patients received 12 neurectomies by the plantar longitudinal approach. All operations were performed by one of two specialist foot and ankle consultant surgeons in our institute. The hospital ethics committee approved this study, as well as the internal review board, and informed consent was obtained from all patients.

The medical notes of each patient, which included pre- and postoperative notes, radiographs, and pathology reports, were retrospectively reviewed. The main demographic characteristics are shown in Table 1. Visual analogue scale (VAS) scores, American Orthopedic Foot and Ankle Society (AOFAS) scores, and the Foot and Ankle Ability Measure (FAAM) for activities of daily living were used to evaluate the outcomes and satisfaction. The AOFAS scores include pain, autonomic activity, maximum walking distance, ground walking, and abnormal gait. The FAAM provides essential details of patient-reported clinical function during activities of daily living and sports. The FAAM has been used for its validity, reliability, and responsiveness. A questionnaire called appearance index (AI) was also used to assess the influence of foot appearance on the quality of life. The AI consists of a questionnaire with five items that estimate the impact of foot appearance regarding the daily activities at public places, leisure, sexual life, work or school, and personal relationships, with four responses scored from 0 to 3, where 0 is the best score [14]. The complications were recorded during the follow-up.

### Statistical analysis

Statistical analysis was performed by the same author using the IBM SPSS version 22 software for Windows (SPSS Inc.). Results are reported as mean ± standard deviation. The Mann–Whitney U-test was used to compare the VAS, AOFAS, FAAM and AI scores of the two groups. A value of *p* < 0.05 was considered statistically significant.

## Results

The mean duration of the follow-up in both groups was 28.9 ± 12.9 months (range, 15–72 months). The dorsal approach group was composed of 3 males and 5 females with an average age of 46.38 ± 12.93 years, and the plantar approach group was composed of 2 males and 10 females with an average age of 50.00 ± 13.48 years. One neuroma was located in the first interspace in the plantar approach group, while others were located in the second or third interspace. Both the second and third interspaces were involved in one patient (Table 1).

**Table 1:** Patient Demographics

	Dorsal Group	Plantar Group
Variable	n = 8	n = 12
Average age, years	46.38 ± 12.93	50.00 ± 13.48
Duration of follow-up, months	31.5 ± 8.35	27.08 ± 15.34
Male, n(%)	3(37.5)	2(16.7)
Female, n(%)	5(62.5)	10(83.3)
Laterality, n (%)		
Left	5(62.5)	6(50)
Right	3(37.5)	6(50)
Location of neuroma,n(%)		
First interspace	0(0)	1(8.3)
Second interspace	4(50)	4(33.3)
Third interspace	3(37.5)	7(58.4)
Second and third interspace	1(12.5)	0(0)

All the histological results of neurectomy were confirmed by a pathologist and identified as Morton’s neuroma. The postoperative mean VAS pain scores were significantly reduced compared with the preoperative scores in both groups. Moreover, the postoperative mean AOFAS and FAAM scores significantly improved in both groups. No significant difference on postoperative VAS pain scores, AOFAS scores and FAAM scores was found between the two groups, but significant difference on AI scores was found between the two groups (Table 2).

**Table 2:** Clinical Variables of the Dorsal Group and Plantar Group

Variable	Dorsal Group	Plantar Group	P value
Preoperative VAS score	5.25 ± 0.71	4.83 ± 0.72	
Postoperative VAS score	1.00 ± 0.76	0.67 ± 0.65	0.310
Preoperative AOFAS score	65.00 ± 5.93	65.58 ± 4.44	
Postoperative AOFAS score	90.38 ± 4.78	94.42 ± 2.27	0.053
Preoperative FAAM score	72.38 ± 4.60	72.42 ± 2.97	
Postoperative FAAM score	92.75 ± 3.28	94.08 ± 3.03	0.392
Postoperative AI score	8.62 ± 1.06	1.58 ± 0.67	0.000

VAS = visual analogue scale

AOFAS = American Orthopedic Foot and Ankle Society

FAAM = Foot and Ankle Ability Measure

AI = Appearance Index

Few complications were reported in both groups. Two cases suffering from postoperative numbness and paraesthesia were reported in the dorsal group, but none of the two was subjected to another surgery to solve these problems. No patient complained about scar problems in the plantar group.

## Discussion

Plantar approach and dorsal approach are mostly used to perform the surgery to treat the Morton’s neuroma. In the present study, both the plantar approach and dorsal approach resulted in satisfactory results. Nevertheless, the plantar approach showed better results on the appearance index than the dorsal approach.

Morton’s neuroma is a multifactorial paroxysmal neuralgia of one or more intermetatarsal spaces (typically the second and the third space), occurring where the plantar digital nerve divides to supply the adjacent sides of the toes [1]. Its aetiology, incidence, and prevalence are unknown [3]. Ultrasound and magnetic resonance imaging (MRI) scans are widely used to diagnose Morton’s neuroma [6, 7]. In our patients, 95% of the neuromas were found by ultrasound, while 85% were detected by MRI. Thus, ultrasound was preferred in this study to diagnose Morton’s neuroma in the outpatients.

In 1940, Betts [15] was the first reporting the successful neurectomy results in treating Morton’s neuroma. From that moment on, neurectomy has been the standard surgical technique in the treatment of Morton’s neuroma. Many different approaches have been reported, including dorsal approach, plantar longitudinal approach, plantar transverse approach, and distal interdigital approach. The dorsal approach and the plantar longitudinal approach are those used most often [16–19].

The supine position is the one facilitating the surgery using the dorsal approach, but more structures must be dissected to reach the nerve, including the intermetatarsal ligament [20]. Mann and Reynolds [21] reported excellent or good results in 86% of the cases, while failure or poor results in 14%. Giannini et al. [10] performed a study involving 63 patients who received neurectomy using the dorsal approach for the treatment of severe pain and reported that the clinical outcome was excellent or good in 78% of the cases, fair in 19%, and poor in 3%. Disadvantages of the dorsal approach include amputation neuroma, inadequate resection of the digital nerve and damage to the cutaneous nerves in the web space that may become painful. Friscia et al. [22] reported 8% complications, and Nashi et al. [23] reported 19% complications with this approach.

As regards the plantar approach, the transected nerve ending is located in a well-protected intermuscular space, away from the weight-bearing area. In addition, forefoot splaying is avoided, since the transverse metatarsal ligament is not sectioned [24]. Kundert et al. [25] reported that complications occurred in 7.1% of patients after the plantar approach was used and scar problems were described in 5.2% of patients, including delayed wound healing, hypertrophic scar formation, and inclusion cyst. Jarde O et al. [26] reported 0–27% painful incisional scars in the plantar longitudinal approach, while Nashi et al. [23] reported complications in 8% of the cases. Beskin et al. [27] reported that 39 patients had recurrence of neuropathic pain symptoms, and among them, the pain occurred within 1 year after the planter surgery in 65% of patients, and between 1 and 4 years in the remaining 35%.

To the best of our knowledge, very few publications are available comparing the dorsal approach and the plantar approach for the resection of a painful Morton’s neuroma. Nashi et al. [23] prospectively compared 52 patients alternatively assigned to the dorsal or plantar approach, and found that patients treated by the dorsal approach need less time to return to work, have shorter hospital stay, better subjective satisfaction, and less complications. Akermark et al. [28] concluded that both approaches have comparable results. Wilson and Kuwada [29] retrospectively compared the results and complications of the dorsal approach and plantar approach; 68% of patients in the dorsal group achieved a complete relief of the symptoms, while 100% of patients in the plantar group achieved a complete remission of the symptoms. Complications in the dorsal group include 6 cases of amputation neuromas. No amputation neuromas were reported in the plantar group, but 2 painful scars were reported. Habashy et al. [30] retrospectively compared the plantar approach to the dorsal approach using the FFI and SF-36 outcome assessment. They found no statistically significant differences between the dorsal and plantar approach groups regarding the outcomes and patient satisfaction, as measured by the SF-36 or the FFI. The plantar approach reduces the chance of recurrent neuroma and has a similar incidence of incisional complications.

Our study retrospectively compared the clinical outcomes of neurectomy in the treatment of Morton’s neuroma by the plantar and dorsal approaches using the Visual analogue scale (VAS) scores, American Orthopedic Foot and Ankle Society (AOFAS) scores, and the Foot and Ankle Ability Measure (FAAM) for activities of daily living. The appearance index (AI) was also firstly used to assess the influence of foot appearance on the quality of life. No significant difference on postoperative VAS pain scores, AOFAS scores and FAAM scores was found between the two groups, suggesting that both approaches can be used to perform the operation. Our recommendation is that the surgeon should select the approach based on its experience. Nevertheless, significant difference on the AI scores was found between the groups. No patient complained of scar problems in the plantar group, since the incision was made in the non-weightbearing area. Thus, if a patient has cosmetic needs, a plantar approach is recommended.

The limitations of our study were the following: it was a retrospective study with potential recall bias and a small sample size. The operations were performed by two different surgeons rather than the same one. The investigation of the outcomes was performed during a mid-term rather than long-term follow-up. All patients in our plantar approach group received a longitudinal plantar incision; thus, no comment could be made on the transverse plantar approach.

## Conclusion

Our study demonstrated that both the dorsal and plantar approach resulted in good outcomes. However, the foot appearance after the surgery by the plantar approach had less influence on the quality of life than the dorsal approach. Our recommendation is that surgeons should select the approach they are most familiar with and with which they are most confident in performing. If a patient has cosmetic needs, a plantar approach is recommended.

## Data Availability

The datasets used and/or analysed during the current study are available from the corresponding author on reasonable request.

## Abbreviations

VAS: visual analogue scale
AOFAS: American Orthopedic Foot and Ankle Society
FAAM: Foot and Ankle Ability Measure
AI: Appearance Index
